# Current News

**Published:** 1901-11

**Authors:** 


### Current News.

### INSTITUTE OF DENTAL PEDAGOGICS.

THE ninth annual meeting of the Institute of Dental Pedagogics will convene on Tuesday December 31, 1901, and continue for three days, at the Hotel, Seventh Avenue, Pittsburg, Pa. The usual New-Year's-Day rates can generally be obtained.

This is the only normal school existing in the dental profession. *Come everybody and see it perform.* A partial programme is submitted:

President's Address. By G. E. Hunt, Indianapolis.

Conduct of the Operatory Clinic (a method of keeping records, grades, etc.). By G. V. Black, Chicago.

Executive Work of the Faculty (a symposium). By Drs. Kirk, Patterson, Stubblefield, and Hart.

Metallurgy: How to teach. By Dr. Hodgen, San Francisco.

Class-Room Methods of Teaching (symposium). By Drs. Hoff, Nones, Tenney, and Foster.

Teaching Prosthetic Dentistry. By G. H. Wilson, Cleveland.

Bacteriology: How to teach. By W. R. Blue, Louisville.

Report of Committee on Operative and Prosthetic Technics. By Drs. Week and Hoff.

D. M. CATTELL,
*Chairman Executive Board.*

### COLORADO STATE BOARD OF DENTAL EXAMINERS.

THE Board of Dental Examiners of the State of Colorado will meet in Denver, Tuesday, December 3, 1901, at nine A.M., for examination of applicants for license to practise dentistry in Colorado.

In addition to written and oral examination, applicants must supply their own patients, instruments, and materials, and come prepared to do practical work under the supervision of the Board, which will pass upon suitable selection of cavities.

All applications must be completed prior to December 3.

For application blanks and information, address

H. F. HOFFMAN, *Secretary,*
611 California Building, Denver, Col.

---

### BOARD OF DENTAL EXAMINERS OF PENNSYLVANIA.

THE Board of Dental Examiners of Pennsylvania will conduct examinations simultaneously in Philadelphia and Pittsburg, December 16 to 19, 1901. Apply to the Hon. James W. Latta, Secretary Dental Council, Harrisburg, Pa., for papers and information.

G. W. KLUMP,
*Secretary.*

---

### PENNSYLVANIA STATE DENTAL SOCIETY.

AT the annual meeting of the Pennsylvania Dental Society, held at Ligonier, July 10 to 12, 1901, the following officers were elected:

President, M. H. Cryer; First Vice-President, R. H. Nones; Second Vice-President, J. A. Libbey; Recording Secretary, C. V. Kratzer; Corresponding Secretary, V. S. Jones; Treasurer, R. H. D. Swing.

*Executive Committee.*—R. H. Nones, Grant Mitchel, H. M. Beck.

V. S. JONES,
*Corresponding Secretary.*

BETHLEHEM, PA.

### MISSISSIPPI VALLEY MEDICAL ASSOCIATION.

The twenty-seventh annual meeting of the Mississippi Valley Medical Association adjourned at Put-in-Bay, after a most successful session, on the morning of the 14th, out of respect to our martyred President. The following officers were elected for the ensuing year:

President, S. P. Collings, M.D., Hot Springs, Ark.; First Vice-President, J. C. Culbertson, M.D., Cincinnati, Ohio; Second Vice-President, Paul Paquin, M.D., Asheville, N. C.; Secretary, Henry Enos Tuley, M.D., Louisville, Ky.; Treasurer, Thos. Hunt Stucky, M.D., Louisville, Ky.; Chairman Committee of Arrangements, A. H. Cordier, M.D., Kansas City, Mo.

The twenty-eighth annual meeting will be held at Kansas City, Mo., October, 1902.

Henry Enos Tuley,
*Secretary.*

---

### COLORADO STATE DENTAL ASSOCIATION.

At the annual meeting of the Colorado State Dental Association, held in the Brown Palace Hotel, Denver, July 9, 10, and 11, the following Officers were elected for the ensuing year:

President, J. Stewart Jackson, Denver; Vice-President, Theodore Ashley, Canon City; Secretary, W. A. Brierley, Denver; Treasurer, Wm. Smedly, Denver.

The next annual meeting will be held in Colorado Springs during June, 1902.

W. A. Brierley,
*Secretary.*

70 Barth Block, Denver, Col.

